# High correlation between Framingham equations with BMI and with lipids to estimate cardiovascular risks score at baseline in HIV-infected adults in the Temprano trial, ANRS 12136 in Côte d’Ivoire

**DOI:** 10.1371/journal.pone.0177440

**Published:** 2017-06-05

**Authors:** Calixte Ghehi, Delphine Gabillard, Raoul Moh, Anani Badje, Gérard Menan Kouamé, Eric Oouttara, Hugues Ahibo, Jean Baptiste N’Takpé, Jérôme Lecarrou, Serge Paul Eholié, Xavier Anglaret, Christine Danel

**Affiliations:** 1 Programme PACCI - ANRS research site, Abidjan, Côte d’Ivoire; 2 Inserm U1219, Université de Bordeaux, Bordeaux, France; 3 Unité de Soins Ambulatoire et de Conseil (USAC), Abidjan, Côte d’Ivoire; 4 Service des Maladies Infectieuses et Tropicales, CHU de Treichville, Abidjan, Côte d’Ivoire; 5 Centre de Recherche et Diagnostic sur le SIDA, (Cedres) CHU de Treichville, Abidjan, Côte d’Ivoire; Fundacao Oswaldo Cruz, BRAZIL

## Abstract

**Context:**

Data on cardiovascular risk (CVR) score among HIV-infected patients in sub-Saharan Africa are scarce. Our first objective was to compare the CVR score of Framingham utilizing BMI and lipids at baseline, and secondary to assess evolution of CVR score over time at Month 30 in the Temprano trial.

**Methods:**

HIV-infected adults with CD4 <800/mm^3^ without criteria for initiating ART were included and followed for 30 months in the Temprano trial, which assessed the benefits and risks of early antiretroviral treatment (ART) vs deferred ART. CVR score was estimated at baseline and Month-30 using Framingham equations with either BMI or lipids and classified as high (>20%), moderate (10–20%), and low risk (<10%). At baseline, we compare these two estimations utilizing the Pearson correlation test and analyze the increasing CV risk score over time by Proportional odds cumulative logit models for people attending the Month-30 (M30) visit.

**Results:**

Among the 2056 patients, 78% were women, median age was 35 years, and median CD4 count was 464/mm^3^, 6.8% were obese, 6.3% had hypertension, 7.8% were smokers (1.8% women, 26.8% men), 19% had Total Cholesterol (TC) >5mmol/L, and 1% diabetes at baseline. At baseline the concordance between the two Framingham equations was excellent (r = 0.95; p<0.0001). Among the 1700 patients who attended M30 visit and with available data, 1.3% had a high CV risk score at baseline and 3.1% at M30 visit using Framingham equation with BMI. Adjusted odds ratio (aOR) of being at a higher CV risk score at M30 visit compared to a higher CV risk score at M0 visit was 1.35 (CI 95% 1.17–1.57). Stratified by sex, the increasing CV risk score was OR 1.73 (CI 95%: 1.30–2.29) for women and OR 1.24 (CI 95%: 1.02–1.50) for men. Early ART was not associated with an increasing CV risk score (p = 0.88). Results for the 1422 patients with Framingham equation using lipids were similar.

**Conclusion:**

In a large trial evaluating early ART for HIV infection in Côte d’Ivoire, Framingham equation with BMI and lipids were highly correlated and CV risk score increases over time. Early ART was not significantly associated with this increasing CV risk score.

## Introduction

Cardiovascular disease (CVD) is the leading cause of death worldwide. More than 75% of the 17.5 million deaths from CVD occur in low- and intermediate-resource settings [1]. Sub-Saharan Africa is the region most affected by the Acquired Immuno Deficiency Syndrome (AIDS) epidemic globally, with 25.8 million people living with HIV (PLWHIV) in 2014 [2]. Widespread use of antiretroviral therapy (ART) has favorably changed outcomes for PLWHIV in this region, resulting in longer lifespans [3]. However, some of these drugs can contribute to lipid disturbances [4, 5] leading to a potential increase in the prevalence of CV risk factors [6–8]. The lengthening of life expectancy with ART, coupled with the role of HIV itself in increasing CV risk [9, 10], calls for special attention to these risk factors in PLWHIV.

The severity of CVD, as a chronic and sometimes fatal illness, stresses the importance of its prevention and detection. But what tools exist in countries with limited resources? The Framingham equation, a simple tool, was developed and validated in a large cohort in the US and Europe [11], where it evolved over time to include lipid measurements as a risk factor [12, 13]. Several studies estimated the CV risk specifically in large cohorts of HIV-infected adults, taking ART use into account [14]. However, there has been no validation of this Framingham score in Africa, and few studies exist on this topic.

In the context of increased ART availability since the early 2000s and recommendations for early treatment [15–17], estimating CV risk with and without ART is of great importance. Our study is nested in the Temprano ANRS 12136 trial, in Abidjan, Côte d’Ivoire, where ART-naive patients were randomized to receive ART immediately at enrolment or deferred ART, with initiation guided by World Health Organization (WHO) recommendations.

Our first objective was to estimate the concordance between Framingham equation with BMI and the Framingham equation with lipids, in order to improve the assessment of CV risk score in African settings where lipids measurements are scarce.

Our secondary objectives were to estimate the evolution of CV risk factors between M0 and M30, the increasing CV risk score using the Framingham equation with lipids or BMI between M0 and month 30 for patients attending the M30 visit, and assess the factors associated with a moderate or high CV risk score at M0 and M30.

## Methods

### Patients

All the participants analyzed in the Temprano trial were included in this study. The design and rationale of the Temprano trial have been described in detail elsewhere [16]. Briefly, Temprano is a multi-center randomized open-label trial assessing the benefits and risks of two interventions in HIV infected participants with high CD4 cell counts: early ART initiation and 6-month isoniazid preventive therapy (IPT). The trial was launched in March 2008 in 9 clinical centers in Abidjan, Côte d’Ivoire, and ended in December 2014. The trial protocol was approved by the institutional review board of the French Research Agency on AIDS and viral hepatitis (ANRS, Paris) and by the Côte d’Ivoire National Ethics Committee. It has been registered on clinicaltrials.gov under the identifier NCT00495651. All patients signed a written consent before inclusion.

The main inclusion criteria were: HIV-1 or dual HIV-1/2 -infected adults; signed informed consent; absence of ongoing active TB; CD4 count ≤800/mm^3^; and no criteria to start ART immediately, according to the most recent WHO guidelines. Participants were randomized into one of four arms: (i) Immediate ART, (ii) Deferred ART, (iii) Immediate ART plus 6-month IPT, and (iv) Deferred ART plus 6-month IPT. Immediate ART consisted of starting ART at enrollment irrespective of CD4 count and clinical stage. Deferred ART consisted of starting ART when WHO clinical and immunological criteria for ART initiation were met. WHO recommendations evolved overtime during the trial (from March 2008 to Nov 2009, patients started ART according to WHO 2006 [18], from Dec 2009 to July 2013, according to WHO 2010 [19], and from August 2013 to Dec 2014, according to WHO 2013[20])

The trial enrolled 2076 patients who were followed for 30 months. The main outcome was the occurrence of a new episode of severe morbidity, including AIDS-defining diseases, non-AIDS defining severe bacterial diseases, non-AIDS defining cancers, and any event leading to death.

### Follow up

At enrolment (M0), we performed a clinical examination to obtain patient weight, height, waist size, and blood pressure, and we performed chest radiography. Data on previous medical events, socio-demographic characteristics, occupation, socio economic level, level of schooling, and use of tobacco and alcohol were collected using questionnaires. We collected blood samples for analysis of blood cell count, CD4 cell count (True Count^®^ technique, FACScan^®^, Becton Dickinson), serum transaminases, serum creatinine, and serum glucose. Total cholesterol, high-density lipoprotein (HDL) cholesterol, low-density lipoprotein (LDL) and triglycerides levels were measured with Cobas Integra 400 (Roche Diagnostics). We measured plasma HIV-1 RNA (real-time PCR, Taq Man technology ABI Prism 7000, Applied Biosystems, detectability 100 copies/mL) at M0 and every 6 months, except lipid and glucose measurements were obtained only at M0 and at M30. Patients were asked to be fasting when they came to the hospital. Preferred first-line ART was a fixed-dose combination of tenofovir disoproxil fumarate (TDF) 300mg and emtricitabine (FTC) 250 mg (Truvada^®^, Gilead) plus efavirenz (EFV) 600 mg (Stocrin^®^, MSD). Patients with contra-indications to efavirenz or women who were not using hormonal contraception were given either Truvada^®^ plus zidovudine 300 mg or Truvada^®^ plus lopinavir/ritonavir 400/100 mg (Kaletra^®^, Abbott), Patients were asked to return for scheduled trial visits at Day 8, M1, M2, M3, and every three months thereafter. Standardized questionnaires were used to record baseline and longitudinal data.

Patients were defined as lost to follow up if their last contact was prior their scheduled 30 months visit, they were not known to be dead, and no further information on their vital status was available by March, 15^th^ 2015.

### Definitions of cardiovascular risk

CV risk factors have been defined by the National Cholesterol Education Program (NCEP ATP III) [21], the Joint National Committee on Prevention, Detection, Evaluation and Treatment of High Blood Pressure (JNC7) [22] and the American Diabetes Association [23]. Based on these recommendations, we defined CV risks factors in our study as: (i) Advanced age (≥45 years old for men and ≥55 years old for women); (ii) Overweight, defined as BMI 25–29.99 kg/m^2^ or obesity BMI≥ 30 kg/m^2^. Height and weight were measured once using a stadiometer and digital balance, respectively. Readings were recorded to the nearest 0.5 centimetre and the nearest 0.1 kg. Participants were weighed and measured without shoes and wearing only light clothing; (iii) Abdominal waist circumference ≥88 cm for women and ≥102 cm for men; (iv) Total cholesterol ≥5 mmol/L, low HDL cholesterol (<1.2 mmol/L for women and <1.0 mmol/L for men); (v) Triglycerides ≥1.7 mmol/L; (vi) Criteria used for the diagnosis of hypertension were proposed by the WHO/International Society of Hypertension using the average brachial systolic BP of 140mmHg or higher, average brachial diastolic BP of 90 mmHg, or use of anti-hypertensive drug at inclusion (M0) or at M30; (vii) Diabetes, defined as repeated fasting glucose ≥1.26 g/l (7.0 mmol/L) or glucose ≥2g/l (11.1 mmol/L) at all times, or treatment for diabetes. We defined a smoker as smoking >1 cigarette per day. We assumed that smoking status was unchanged from M0 to M30.

We used two Framingham risk equations to quantify the estimated 10-year absolute CV risk for each individual at M0 and M30. For both equations, variables used for calculating the Framingham risk score included age, sex, smoking status, prevalent diabetes, use of anti-hypertensive drugs, and systolic blood pressure. In the first equation, BMI was used [24], and in the second equation, total and HDL cholesterol were used [12]. We defined groups of CV risk: low risk (<10%), moderate (10–20%), and high (>20%).

Life conditions were a composite variable combining access to water, electricity, and refrigerator. We defined life conditions as bad if none of these were at home, moderate if water and electricity, and good if water, electricity, and refrigerator were all available at home.

CV manifestations taken into account in the Framingham Equation were coronary death, myocardial infarction, coronary insufficiency, angina, ischemic stroke, hemorrhagic stroke, transient ischemic attack, peripheral artery disease, and heart failure.

### Statistical analysis

None of the patients included in this study presented with CVD at baseline.

We described (i) baseline characteristics stratified by sex,(ii) prevalence of CV risk factors (age, gender, obesity, overweight, waist circumference, diabetes mellitus, TC, HDL cholesterol, triglycerides, tobacco, high blood pressure (HBP)) at M0 and M30 visit. For patients who attended the M30 visit, we compared the prevalence of CV risk factors between M0 and M30 for women and men with McNemar test, Chi^2^ test, or Fisher’s exact test.

The Framingham risk score using BMI or lipid was calculated at M0 and M30. Patients with biological missing data were excluded from this analysis. We estimated the concordance between the two Framingham equations using the Pearson correlation test at baseline. Odds Ratio with confidence interval (CI 95%) and p-value were calculated using proportional odds cumulative logit models with random effects to compare the risk of having a higher CV risk score (Framingham score in 3 classes) between M0 and M30. So we compare the high CV risk score vs moderate or low; and the high or moderate CV risk score vs low.

First, we did adjusted models on sex and ART arms, and then stratified models on sex, and then on sex and ART arms to estimate the CV risk score for the different populations.

In univariable and multivariable analysis with logistic regression model we analyzed the association between high or moderate CV risk score at inclusion, at month-30, and with higher CV risk score at M30 as estimated by the BMI Framingham equation with the following variables in the population of patients attending a visit in M30 using SAS^®^ version 9.2 (SAS institute Inc., Cary, North Carolina, USA): education level, professional activities, matrimonial status, conditions of life, WHO stage, CD4 cell count, HIV viral load, and duration of ART exposure. These analysis were planned after the last version of the protocol.

The protocol was approved by the Ivory Coast National Ethics Committee for Health Research. The sponsor (the French National Agency for Research on AIDS and Viral Hepatitis [ANRS]) had no role in the conduct of the study or the interpretation of the data.

Merck Sharp & Dohme donated Stocrin (efavirenz) and Gilead Sciences donated Truvada (tenofovir–emtricitabine) for all participants in the study; these companies had no other role in the study. Nouvelle Pharmacie de Santé Publique of Ivory Coast provided all other antiretroviral drugs, with support from the U.S. President’s Emergency Plan for AIDS Relief.

## Results

### Patient characteristics

**Fig 1:**
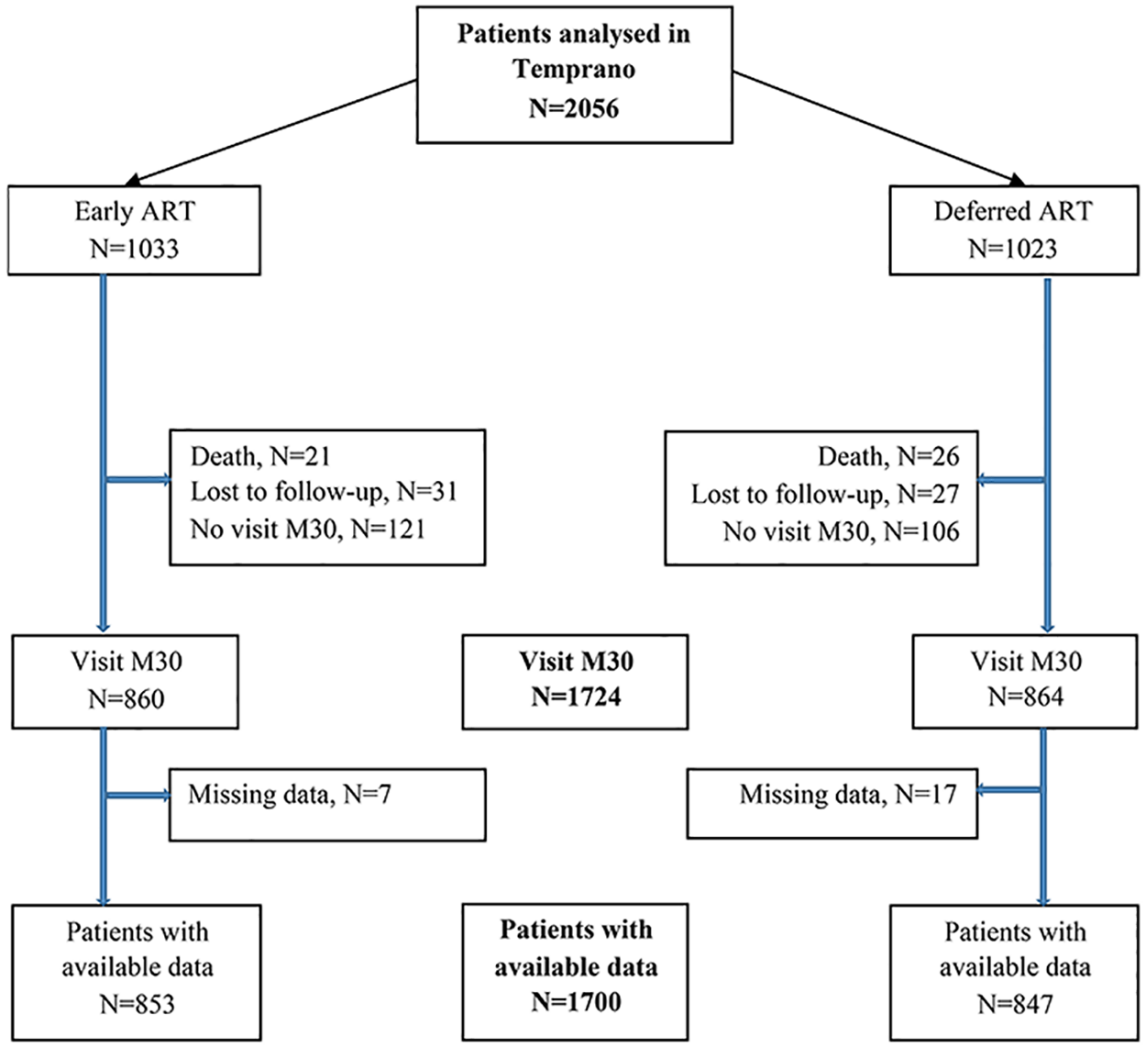
Flow chart of the Temprano trial, (N = 2056).

Among the 2056 patients analyzed, 1724 patients attended the M30 visit. At study termination, 47 patients (2%) were known to have died, 58 (3%) were considered to have been lost to follow-up, and 227 (11%) did not attend the M30 visit, with no significant differences among the deferred ART and immediate ART, arms of the study. (Fig 1).

In Table 1, baseline characteristics of the 2056 patients are detailed by sex. The majority of participants were female (78.5%). In this cohort, women were less educated than men and worked more often in an informal sector. Men were older, more often hypertensive, and more frequently smokers than women. Women were more often overweight or obese and had lower HDL cholesterol levels. Protease Inhibitors (PI) were more often prescribed for women (efavirenz was possible only for women who accepted hormonal contraceptive).

**Table 1:** Baseline characteristics for patients included in Temprano trial; N = 2056.

	All	Women	Men	p
*Baseline*				
Age in years, median (IQR)	35 [30–42]	34 [29–40]	40 [34–46]	*0*.*0001*
Schooling, n (%)				
Never	521 (25.3)	455 (28.2)	66 (14.9)	*0*.*0001*
Primary	593 (28.8)	517 (32.0)	76 (17.2)	
Secondary	681 (33.0)	469 (29.1)	212 (48.0)	
Superior	261 (12.7)	173 (10.7)	88 (19.9)	
Activity sector, N (%)				*0*.*0001*
No activity	604 (29.4)	532 (33.0)	72 (16.3)	
Public or private	501 (24.4)	254 (15.7)	247 (55.9)	
Informal	951 (46.2)	828 (51.3)	123 (27.8)	
No activity,	604 (29.4)	532 (33.0)	72 (16.3)	
Living conditions*, N (%)				
Bad,	358 (17.4)	282 (17.5)	76 (17.2)	*0*.*90*
Moderate	907 (44.1)	708 (43.9)	199 (45.0)	
Best	791 (38.5)	624 (38.6)	167 (37.8)	
WHO clinical stage, n (%)				
stage *1*	1321 (64.2)	1045 (64.7)	276 (62.4)	*0*.*54*
stage *2*	537 (26.1)	418 (25.9)	119 (26.9)	
stage *3*	187 (9.1)	144 (8.9)	43 (9.7)	
CD4 count, cell/mm^3^ Median (IQR)	464 [369–573]	466 [373–573]	457 [358–578]	*0*.*71*
Plasma HIV-1 RNA, log_10_ copies/ml, median (IQR)	4.6 [3.9–5.2]	4.5 [3.9–5.1]	4.9 [4.3–5.4]	*0*.*0001*
Body mass Index (Kg/m^2^)				
Median [IQR]	22.4 [20–25]	22.7 [20.3–25.7]	21.5 [19.8–24.2]	*0*.*0001*
Strata				
Thin <18.5	196 (9.5)	156 (9.7)	40 (9.1)	*0*.*0001*
Normal 18.5–24.9	1296 (63.0)	978 (60.6)	318 (71.9)	
Overweight 25–29.9	425 (20.7)	354 (21.9)	71 (16.1)	
Obese ≥30	139 (6.8)	126 (7.8)	13 (2.9)	
High Blood pressure ^μ^	130 (6.3)	93 (5.8)	37 (8.4)	*0*.*04*
TG > 1.7 mmol/l, N(%)^μ^	164 (8.0)	103 (6.4)	61 (13.9)	*0*.*0001*
TC > 5 mmol/l, N(%)^μ^	389 (19.0)	317 (19.7)	72 (16.4)	*0*.*11*
HDL-c < 1.2 mmol/l,N(%)^μ^	953 (57.6)	953 (60.0)	153(50.7)	*0*.*003*
Diabete mellitus, N(%)	21 (1.0)	15 (0.9)	6 (1.4)	*0*.*42*
Smokers, N(%)	161 (7.8)	33 (2.0)	128 (29.0)	*0*.*0001*
Temprano Arm randomisation				
Early ART	1033 (50.2)	823 (51.0)	210 (47.5)	*0*.*19*
Deferred ART	1023 (49.8)	791 (49.0)	232 (52.5)	
*Follow-up*				
Never started ART, n (%)	426 (20.7)	326 (20.2)	100 (22.6)	*0*.*0001*
First-line ART regimen, n (%)				
* TDF–FTC plus EFV*	1145 (55.7)	822 (50.9)	323 (73.1)	
* TDF–FTC plus LPV/r* ¥	404 (19.7)	388 (24.0)	16 (3.6)	
* Other* §	81 (3.9)	78 (4.8)	3 (0.6)	

**^μ^: missing data** (for women, n = 2 for BP, n = 6 for viral load, and HDL, n = 5 for Triglyceridemia (TG), and Total cholesterol (TC), for men, n = 2 for viral load, n = 3 for TG and TC, n = 4 for HDL)

**$**: p value compare the prevalence of events between men and women (x^2^ test or fisher test)

**BMI**: Body Mass Index

**TG**: Triglyceridemia

**TC**: Total Cholesterol

**HDL-c**: Serum High Density Lipoprotein cholesterol

**ART**: Antiretroviral therapy

**IQR**: interquartile range

**N**: number

**%** percentage

*: Living conditions (see the Methods section)

**NRTI**: Nucleosidic reverse transcriotase inhibitor

**NNRTI**: Non Nucleosidic reverse transcriptase inhibitor,** 99,7% was Efavirenz

**LPV/r**: Lopinavir/ ritonavir

^¥^ Among the 377 patients who started ART with TDF–FTC plus LPV/r, the reason for not receiving an EFV-based regimen was dual infection with HIV-1 and HIV type 2 (15 patients), a history of prevention of mother-to-child transmission with nevirapine (38), declining to use effective contraception (308), and other reasons (16).

^§^ Other regimens were TDF–FTC–zidovudine (ZDV) (81 patients), ZDV–lamivudine (3TC)–LPV/r (25), ZDV–3TC–EFV (3), ZDV–3TC–nevirapine (3), didanosine–3TC–EFV (1), stavudine (D4T)–3TC–EFV (1), D4T–3TC–LPV/r (1), and 3TC–abacavir–LPV/r (1).

The median time on ART was 15 months (IQR: 3–15) in the deferred ART and 29.9 months (IQR: 29.9–30) for the Early ART Arms.

### Follow-up

Patients were followed for 4757 person-years (PY). When we compared baseline characteristics of the 332 participants who did not attend the M30 visit to the 1724 who did attend this visit, we found a similar proportion of women (78.6% vs 78.0%; p = 0.81), and WHO stage (Stage 1: 64.6% vs 62.3%; p = 0.72). Median CD4 cell count (464/mm3 vs 466/mm3; p = 0.41) and median HIV viral load (4.6 log10/ml vs 4.7 log10/ml; p = 0.40) were also comparable. The participants who attended the M30 visit were older (median years (IQR) 35(30–42) vs 33 (27–40); p = 0.0002), less often smokers (124 (7.2%) vs 37 (11.1%); p = 0.01), and more often overweight or obese (492 (28.5%) vs 72 (21.7%)). At baseline, the proportion of patients with a high, moderate, and low level of CV risk, as measured with the Framingham equation using BMI, was 1.0%, 4.2% and 94.7%, respectively for the 334 patients who did not attend the M30 visit. These values were 1.5%, 3.1%, and 95.7% for the 1724 participants who attended the last visit (p = 0.47).

The Framingham equation with BMI was calculated for 1700 patients and the equation with lipids was calculated for 1422 patients. Missing data were mainly on HDL and TC (278 patients). The main characteristics for these two populations (Sex, Age, CD4 count at baseline, BMI) were not statistically different (data not shown).

### Evolution of CV risk factors between baseline and M30 by sex

**Fig 2:**
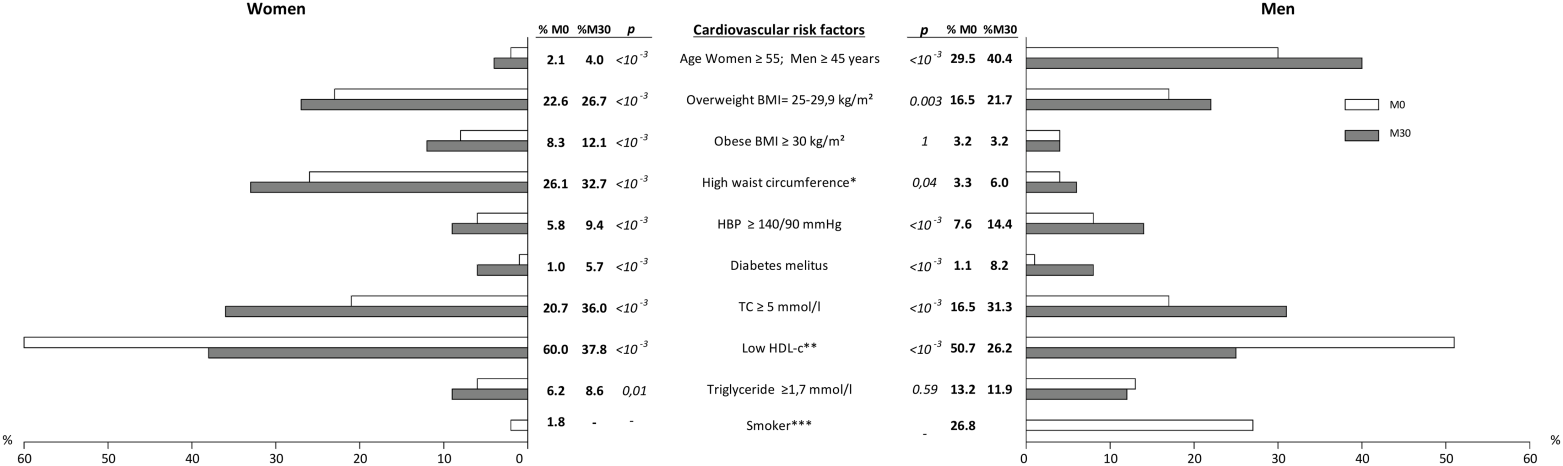
Changes in prevalence of different CV risk factors according to sex in patients with available data at M0 and M30, Temprano trial (N = 1724). * High waist circumference: ≥88 cm in women and ≥102 cm in men. **TC: total cholesterol. *** Low HDL-c: High density lipoprotein cholesterol, <1.2 mmol/l in women and <1 mmol/l in men. ****Smoker is defined as an individual who smokes at least one cigarette per day. BMI: body mass index; HBP: high blood pressure; HDL-c: high density lipoprotein cholesterol; P: p-value of Chi 2 test.

Fig 2 shows the evolution of CV risk factors between baseline and M30 for men and women. For women, all the CV risk factors increased significantly (age, overweight status, waist circumference, high blood pressure, diabetes mellitus, TC, and triglycerides).

For men, only the following CV risk factors did not increase significantly from baseline to M30: proportion of obesity, and triglycerides. Compared to women, men were more often smokers (26.5% vs 1.8%; p = 0.0001). For both, HDL-cholesterol has decreased.

### Comparison between Framingham using BMI and Framingham using lipids at baseline

**Fig 3:**
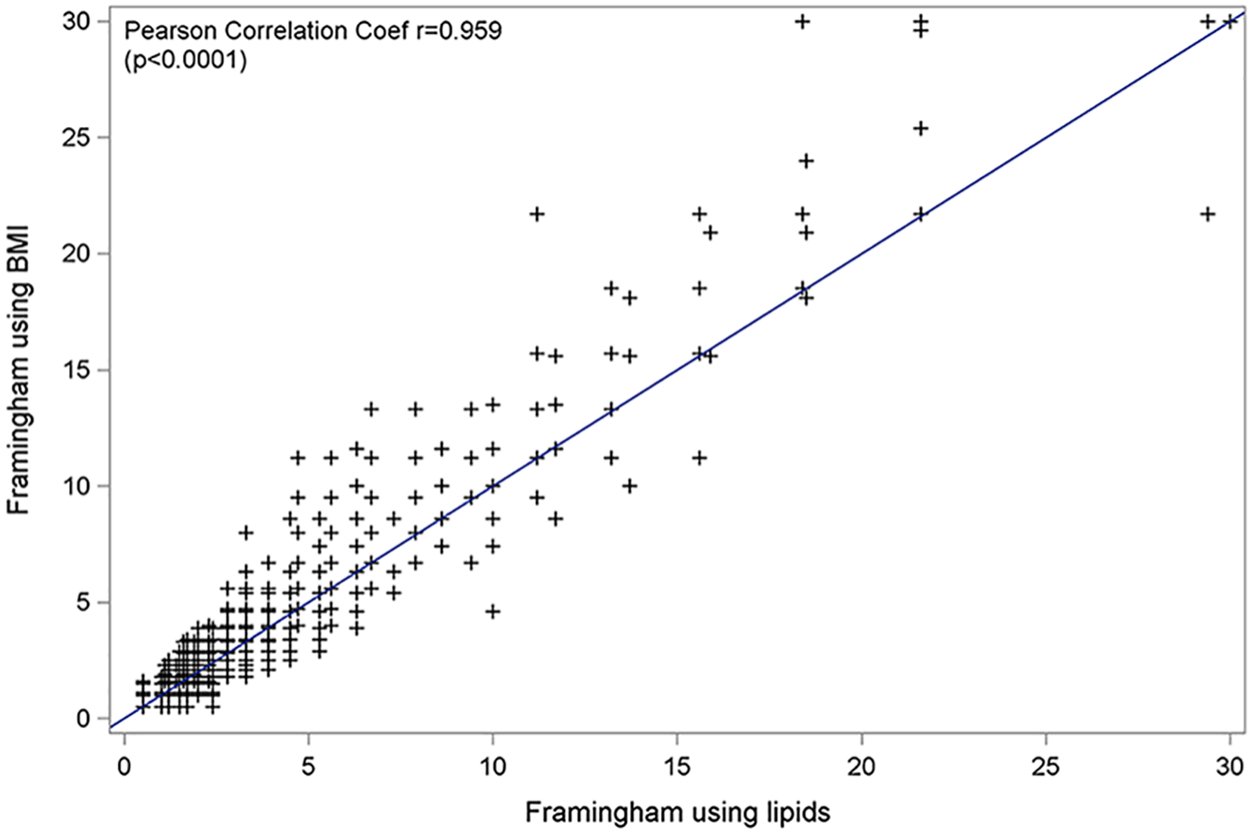
Agreement between the Framingham equations using lipids and BMI; Temprano trial (N = 1422).

Fig 3 shows the concordance between CV risks score estimated with the Framingham equation using BMI and the Framingham equation estimated using lipids. We used the Pearson correlation test to estimate the concordance for all 1422 patients without missing data at baseline which demonstrated an very strong correlation (r = 0.95 p <0.0001) between the two Framingham equations using BMI and lipids.

### Evolution of CV risks scores with the Framingham equation using lipids and BMI between baseline and M30 (Tables 2 and 3)

**Table 2:** Evolution of the CV risk score with Framingham equation (with lipids) between M0 and M30. Proportional odds cumulative logit model, N = 1422*.

Framingham equation (lipids)	Level of CV risk¤	M0	M30	OR (CI 95%)^£^ *M30 vs M0*	*p*
		N (%)	N %		
All patients				1.28 (1.05–1.57) **	*0*.*01*
	Low	1352 (95)	1334 (94)		
	Moderate	59 (4)	57 (4)		
	High	11 (2)	31 (2)		
Model Stratified by sex					
Women				1.57 (1.09–2.25)	*0*.*01*
	Low	1098 (98)	1085 (97)		
	Moderate	24 (2)	27 (2)		
	High	--	10 (1)		
Men				1.16 (0.91–1.49)	*0*.*22*
	Low	254 (85)	249 (83)		
	Moderate	35 (12)	30 (10)		
	High	11 (4)	21 (7)		
Model Stratified by sex and by Temprano ART Arms:					
Men					
Deferred ART	Low	109 (87)	106 (85)	1.21 (0.86–1.68)	*0*.*27*
	Moderate	10 (8)	13 (10)		
	High	6 (5)	6 (5)		
Early ART	Low	145 (83)	143 (82)	1.15 (0.81–1.62)	*0*.*42*
	Moderate	25 (14)	17 (10)		
	High	5 (3)	15 (9)		
Women					
Deferred ART	Low	456 (98)	449 (96)	1.73 (1.04–2.88)	*0*.*03*
	Moderate	10 (2)	14 (3)		
	High	0 (0)	3 (1)		
Early ART	Low	642 (98)	636 (97)	1.45 (0.87–2.41)	*0*.*14*
	Moderate	14 (2)	13 (2)		
	High	0 (0)	7 (1)		

*HDL cholesterol or Total Cholesterol is missing for 2 patients at baseline and 276 patients at months 30 (274 HDL: 21 for early ART and 253 for differed ART; 268 CT: 20 for early ART and 248 for differed ART) and therefore it was not possible to estimate the Framingham equation with lipids for these patients.

^¤^ Level of CV risk estimated at 10 years with the Framingham equation; Low (< 10%), moderate (10–20%), High (>20%)

^£^ OR of being in a higher CV risk score at month-30 compared to be in a higher CV risk score at baseline.

** OR of being in a higher CV risk score adjusted on Temprano ART arms of randomisation (Early ART or Differed ART) and on sex, effect of Temprano arm was not significant (p = 0.53)

**Table 3:** Evolution of the CV risk score with Framingham equation using BMI between M0 and M30. Proportional odds cumulative logit model, N = 1700.

Framingham equation (BMI)	Level of CV risk score¤	M0	M30	OR (CI 95%)^£^ *M30 vs M0*	*p*
		N (%)	N %		
All patients				1.35 (1.17–1.57)**	*<0*.*001*
	Low	1590 (94)	1556 (92)		
	Moderate	88 (5)	92 (5)		
	High	22 (1)	52 (3)		
Stratified by sex					
Women				1.73 (1.30–2.29)	*0*.*0001*
	Low	1308 (98)	1286 (96)		
	Moderate	28 (2)	32 (2)		
	High	4 (0)	22 (2)		
Men				1.24 (1.02–1.50)	*0*.*03*
	Low	282 (78)	270 (75)		
	Moderate	60 (17)	60 (17)		
	High	18 (5)	30 (8)		
Stratified by sex and Temprano ART Arms					
Men					
Deferred ART	Low	146 (81)	141 (78)	1.19 (0.93–1.52)	*0*.*15*
	Moderate	26 (14)	28 (15)		
	High	9 (5)	12 (7)		
Early ART	Low	136 (76)	129 (72)	1.28 (0.96–1.71)	*0*.*08*
	Moderate	34 (19)	32 (18)		
	High	9 (5)	18 (10)		
Women					
Deferred ART	Low	677 (97)	634 (95)	1.73 (1.19–2.52)	*0*.*003*
	Moderate	17 (3)	19 (3)		
	High	2 (0)	13 (2)		
Early ART	Low	661 (98)	652 (97)	1.72 (1.13–2.64)	*0*.*01*
	Moderate	11 (2)	13 (2)		
	High	2 (0)	9 (1)		

^¤^ Level of CV score estimated at 10 years with the Framingham equation; Low (< 10%), moderate (10–20%), High (>20%)

^£^ OR of being in a higher CV risk score at month-30 compared to be in a higher CV risk score at baseline.

** OR of being in a higher CV risk score adjusted on Temprano ART arm of randomisation (Early ART or Deferred ART) and sex. The effect of ART arms of randomisation was not significant (p = 0.88)

For the 1422 participants who attended M30 visit and had available lipids, the increasing level of risk score at Months 30 compared to M0, adjusted for Temprano ART arms and sex was 1.28 (CI 95% 1.05–1.57). In a model adjusted for sex and Temprano ART arms of randomization, effect of randomization Arms was not significant (p = 0.61). The increasing risk from baseline to M30 for men compared to women was OR 7.01 (CI95% 4.62–10.66) p < .001 in a model adjusted for sex.

When stratified by sex, then the increasing risk between M0 and M30 was 1.57 (CI_95%_: 1.09–2.25) p = 0.01 for women and 1.16 (CI_95%_: 0.91–1.49) for men (p = 0.22). When stratified by sex and Temprano ART arms, then there was a significant increasing risk was for women on deferred ART (1.73 (CI_95%_: 1.04–2.88) p = 0.03) (Table 2).

For the 1700 patients with Framingham equation using BMI, the level of increasing CV risk score was similar between baseline and M30 OR 1.35 (CI _95%_ 1.17–1.57) p < .001. The effect of Temprano ART arms was not significant in an adjusted model (p = 0.88) and the interaction test between sex and visit was of limited significance (p = 0.055). When the model is stratified by sex, the risk was 1.24 (CI _95%_ 1.02–1.50) for men and 1.73 (CI _95%_ 1.30–2.29) for women. When stratified by ART arm randomization, significant increasing risk was for women OR 1.73 (CI_95%_: 1.19–2.52) p = 0.004 on deferred ART and OR 1.72 (CI_95%_ 1.13–2.64) p = 0.01 on early ART. The increasing risk was not statistically significant for men (Table 3)

Among the 1700 participants without missing data, 692 (40.7%) increased their CV risk score between baseline to M30 with a median (IQR) = +1 (0.5–2.0). 659 (38.8%) did not change their CV risk score, and 349 (20.5%) decreased their CV risk score (median (IQR) = -1 (-1.7–0.6)) (S1 Table).

### Factors associated with high CV risk score

#### Variables associated with high or moderate CV risk score at M0

In a multivariable analysis, factors associated with high or moderate CV score at baseline were: worker status (public/private vs no activity (aOR 1.76; 95% CI: 1.07–2.87)); informal vs no activity (aOR = 0.73; 95% CI: 0.46–1.17; p = 0.001); matrimonial status (married vs single (aOR = 3.67; 95% CI: 2.24–6.02); divorced vs single (aOR = 5.12; 95% CI:2.74–9.58; p = 0.0001) (S2 Table).

#### Variables associated with increasing CV risk score at M30 or with moderate and high CV risk score at M30

In a multivariable analysis, factors associated with increasing risk score at Month 30 were workers status (public/private vs no activity; aOR 1.35; 95% CI:1.02–1.78)); informal vs no activity (aOR = 0.93, 95% CI: 0.73–1.19; p = 0.02); matrimonial status (married vs single, aOR = 1.51; 95% CI:1.22–1.87); divorced vs single (aOR = 1.91; 95% CI: 1.36–2.67; p = 0.0002); conditions of life (moderate vs bad (aOR = 0.76, 95% CI:0.58–1.02)); best vs bad (aOR = 0.68, 95% CI: 0.51–0.92; p = 0.04) (S3 Table). In a multivariable analysis the factors associated with high or moderate score at M30 were: worker status (public/private vs no activity (aOR = 1.99; 95% CI: 1.25–3.16)); informal vs no activity (aOR = 0.93; 95% CI: 0.59–1.46; p = 0.0005); matrimonial status (married vs single (aOR = 3.26; 95% CI: 2.09–5.09); divorced vs single (aOR 4.33 95% CI: 2.42–7.73; p = 0.0001)), WHO stage (2 vs 1 (aOR = 1.69; 95% CI: 1.16–2.46), 3&4 vs 1 (aOR = 0.90, 95% CI: 0.45–1.80; p = 0.01)) (S4 Table).

## Discussion

In a context where early ART initiation is recommended for PLWHIV [15–17], we describe (i) the excellent concordance between the Framingham equation using BMI and the Framingham equation using lipids in a sub-Saharan African population (ii) the frequency of different CV risk factors by sex prior to initiation of treatment and at M30, (iii) the association between baseline characteristics and moderate and high CV risk score at M0 and M30, (iv) and the increasing CV risk score between M0 and M30 according to the two Framingham equations.

The evaluation of CV risk score at 10 years has been estimated in other African contexts [25–27], but no assessment of the correlation between the two Framingham equations with BMI and with lipids has been done, to our knowledge. In a context where the measurement of lipids is not usually made in HIV clinics, as in Sub-Saharan Africa, this excellent concordance between the two equations can give a simple way to estimate the CV risk score in HIV-infected people. We show an increasing CV risk score over time but no association between early ART initiation and CV risk score at 10 years as estimated by the Framingham equation at 30 months of follow-up in the Temprano trial. CV risk score increased significantly over time in both the deferred ART and early ART strategies. This result can be partially explained by the fact that, in the deferred ART arms, 79% of patients finally were on ARV treatment at Month 30, even if the median time on ART was significantly lower in the deferred ART. The role of ART in CV risk was previously shown in large cohorts [14], but recent data show the reduction of CVD as a cause of death in HIV-infected patients. ART can raise TC and triglycerides but at the same time is reducing inflammation so that despite appearing to work at cross-purposes, the overall benefit of early ART outweighs the risks [28, 29].

The role of HIV itself in CV risk was shown 10 years ago in structured treatment interruption (STI) studies; HIV infection contributed to increased inflammatory factors (e.g., IL6, TNF…) [10]. If earlier ART is part of the answer to decreasing the CV risk related to this HIV inflammation [30, 31], and if new ART regimens are better tolerated, the fact remains that traditional CV risk factors should be taken into account to reduce CV incidence, such as tobacco use [32].

In our study, the most significant factors involved in increased CV risk between M0 and M30 were age, as expected, but also increased prevalence of diabetes, hypertension, and cholesterol over the 30-month period. In women, increased obesity [33] with an associated increase in abdominal circumference, was itself predictive of the myocardial infarction risk [34, 35]. In men, smoking prevalence was higher than in women, as in others studies [27, 36]. The median age was 34 years old, with low levels of hypertension close to others figures in West African HIV infected adults in others studies [37, 38].

Social dynamics, dietary habits, and genetics can explain the different frequencies of risk factors between men and women, as well as the differences between African and Western countries. Indeed, the HIV epidemic in Sub-Saharan Africa is mostly female [7], and infected women tend to be younger than men [25, 27, 39]. This may explain most of the higher severe risk at 10 years in the Framingham score estimation for men compared to women.

Although the prevalence of risk factors among African PLWHIV has been described for hypertension [38–41] and hypercholesterolemia [25], few authors have shown the evolution of these risk factors over time with and without ART. Age is a factor that can, alone, explain the increased prevalence of some factors as hypertension [8]; however, the use of the most common protease inhibitors in women in our study could also be a factor that can explain the increase of total cholesterol [42, 43].

Our study presents several limitations. At Month-30 visit, we considered only patients who survived, were not lost to follow up, and attended the Month-30 visit. This could have lead to a selection bias. But we compared the cardio vascular risk score at baseline between people who attended the Month-30 visit and those excluded for death, lost to follow up before Month-30 and people who didn’t attend Month-30. And the cardio vascular risk was not significantly different between these two groups. Because the study was not designed to estimate the cardiovascular risk score, we have no trends in smoking at 30 months, then we assumed that the proportion of smokers didn’t change over time for the calculation of the score at Month 30. Our study has a short duration of follow up, only 30 months. Most of the patients are female and young while the Framingham study score has been calculated in a Northern population, mostly male. For physical activity, we don’t have any data on this issue, however, professional activity is an indirect marker of the latter. Indeed, people with formal professional activities are often more sedentary than those in informal activity in Africa, and the association between type of work and the higher risk at 30 months shows in our study.

We chose to estimate only Framingham scores and not the DAD score, because HIV patients were naïve to ART at baseline and we didn’t collect data on family history of CVD. However, our main goal was to assess the evolution over time of the risk and to find a simple tool to estimate the cardiovascular risk more than an evaluation of the score itself. None of the risk equations (Framingham or DAD scores) have been validated in Africa yet.

In conclusion, in a large trial evaluating early ART for HIV infection in Côte d’Ivoire, Framingham equation with BMI and lipids was highly correlated and CV risk score increases over time. Early ART was not significantly associated with this increasing CV risk score.

Framingham score with BMI can thus be more easily used in developing countries. Early ART has shown its effectiveness in improving infectious diseases outcomes for PLWHIV in Sub-Saharan Africa. However, even if the CV risk level estimated in our context is lower than in other continents, it is too often underestimated in Africa. This type of assessment, performed with simple and accessible tools, should be recommended to facilitate the prevention of modifiable CVD risk factors, especially in PLWHIV in areas with limited resources.

## Supporting information

S1 Table Distribution of change in CV risk score between M30 and M0 according to sex in Temprano trial, (N = 1700). (DOCX)

S2 Table Association between patient baseline characteristics and moderate/high CV risk score at M0 in Temprano trial, Framingham with BMI, Abidjan (N = 2038). (DOCX)

S3 Table Association between patient baseline and therapeutic characteristics and increasing CV risk score at M30 in Temprano trial, Framingham with BMI, Abidjan (N = 1700). (DOCX)

S4 Table Association between patient baseline and therapeutic characteristics and moderate/high CV risk score at M30 in Temprano trial, Framingham with BMI, Abidjan (N = 1700). (DOCX)
