# Supplementary material for: High correlation between Framingham equations with BMI and with lipids to estimate cardiovascular risks score at baseline in HIV-infected adults in the Temprano trial, ANRS 12136 in Côte d’Ivoire
Source: PLoS One. 2017 Jun 5;12(6):e0177440. doi: 10.1371/journal.pone.0177440 (PMC5459337; doi:10.1371/journal.pone.0177440)
Supplement: S1 Table — (DOCX) [file pone.0177440.s001.docx]

**S1 Table:** Distribution of change in CV risk score between M30 and M0 according to sex in Temprano trial, (N = 1700).

|  | Total | | Women | | Men | |
| --- | --- | --- | --- | --- | --- | --- |
|  | N (%) | ∆RCV Median (IQR) | N (%) | ∆RCV Median (IQR) | N (%) | ∆RCV Median (IQR) |
| ∆RCV (M30-M0) | 1700 | 0 (0, 0.6) | 1340 | 0 (0, 0.6) | 360 | 0 (0, 2.1) |
| ∆RCV zero | 659 (38.8) | 0 (0, 0) | 535 (40.0) | 0 (0, 0) | 124 (34.5) | 0 (0, 0) |
| ∆RCV positive | 692 (40.7) | 1 (0.5, 2.0) | 527 (39.3) | 0.8 (0.5, 1.6) | 165 (45.8) | 2.4 (1.6, 4.5) |
| ∆RCV negative | 349 (20.5) | -1 (-1.7, -0.6) | 278 (20.7) | -0.8 (-1.3, -0.6) | 71 (19.7) | -1.7 (-3.2, -1.1) |

**RCV:** reference change value; **IQR:** interquartile range
