# Supplementary material for: High correlation between Framingham equations with BMI and with lipids to estimate cardiovascular risks score at baseline in HIV-infected adults in the Temprano trial, ANRS 12136 in Côte d’Ivoire
Source: PLoS One. 2017 Jun 5;12(6):e0177440. doi: 10.1371/journal.pone.0177440 (PMC5459337; doi:10.1371/journal.pone.0177440)
Supplement: S2 Table — (DOCX) [file pone.0177440.s002.docx]

**S2 Table:** Association between patient baseline characteristics and moderate/high CV risk score at M0 in Temprano trial, Framingham with BMI, Abidjan (N=2038).

|  | | **Univariable analysis** | | | **Multivariable analysis** | | |
| --- | --- | --- | --- | --- | --- | --- | --- |
| **Variable** | **Unit** | **OR** | **CI_95%_** | *P* | **aOR** | **CI_95%_** | *P* |
| **Education level** | Primary vs Never | 0.71 | 0.44-1.16 | *0.09* | 0.86 | 0.52-1.42 | *0.15* |
|  | Secondary vs Never | 0.98 | 0.63-1.52 |  | 1.03 | 0.64-1.67 |  |
|  | Superior vs Never | 0.44 | 0.21-0.94 |  | 0.44 | 0.20-0.98 |  |
| **Employment** | Public/Private vs No activity | 1.69 | 1.07-2.67 | *0.002* | 1.76 | 1.07-2.87 | *0.001* |
|  | Informal vs No activity | 0.82 | 0.52-1.29 |  | 0.73 | 0.46-1.17 |  |
| **Matrimonial status** | Married vs Single | 3.89 | 2.39-6.35 | *0.0001* | 3.67 | 2.24-6.02 | *0.0001* |
|  | Divorced vs Single | 5.19 | 2.85-9.47 |  | 5.12 | 2.74-9.58 |  |
| **Living conditions*** | Moderate vs Bad | 1.13 | 0.66-1.90 | *0.85* | - | - |  |
|  | Best vs Bad | 1.16 | 0.68-1.98 |  | - | - |  |
| **WHO stage** | 2 vs 1 | 1.44 | 0.97-2.14 | *0.18* | 1.38 | 0.92-2.08 | *0.28* |
|  | 3 & 4 vs 1 | 1.20 | 0.65-2.21 |  | 1.15 | 0.61-2.16 |  |
| **CD4 (cells/mm^3^)** | ≤500 vs >500 | 1.08 | 0.75-1.57 | *0.65* | - | - |  |
| **Viral load (copies/ml)** | ≤5 vs >5 Log**_10_** | 0.67 | 0.46-0.97 | *0.03* | 0.71 | 0.49-1.03 | *0.07* |

**OR:** odds ratio; **aOR:** adjusted odds ratio; **CI:** confidence interval; **WHO:** World Health Organization; **P:** p-value of the Logistic Model

*see Methods section for definition of living conditions
