# Supplementary material for: High correlation between Framingham equations with BMI and with lipids to estimate cardiovascular risks score at baseline in HIV-infected adults in the Temprano trial, ANRS 12136 in Côte d’Ivoire
Source: PLoS One. 2017 Jun 5;12(6):e0177440. doi: 10.1371/journal.pone.0177440 (PMC5459337; doi:10.1371/journal.pone.0177440)
Supplement: S3 Table — (DOCX) [file pone.0177440.s003.docx]

**S3 Table:** Association between patient baseline and therapeutic characteristics and increasing CV risk score at M30 in Temprano trial, Framingham with BMI, Abidjan (N=1700).

|  | | **Univariable analysis** | | | **Multivariable analysis** | | |
| --- | --- | --- | --- | --- | --- | --- | --- |
| **Variable** | **Unit** | **OR** | **CI_95%_** | *P* | **aOR** | **CI_95%_** | *P* |
| **Education level** | Primary vs Never | 0.99 | 0.76-1.30 | *0.11* | 1.09 | 0.83-1.43 | *0.23* |
|  | Secondary vs Never | 0.91 | 0.71-1.18 |  | 0.98 | 0.75-1.29 |  |
|  | Superior vs Never | 0.68 | 0.49-0.96 |  | 0.74 | 0.51-1.09 |  |
| **Employment** | Public/Private vs No activity | 1.27 | 0.98-1.65 | *0.15* | 1.35 | 1.02-1.78 | *0.02* |
|  | Informal vs No activity | 1.04 | 0.82-1.31 |  | 0.93 | 0.73-1.19 |  |
| **Matrimonial status** | Married vs Single | 1.57 | 1.28-1.94 | *0.0001* | 1.51 | 1.22-1.87 | *0.0001* |
|  | Divorced vs Single | 1.92 | 1.39-2.66 |  | 1.91 | 1.36-2.67 |  |
| **Living conditions*** | Moderate vs Bad | 0.75 | 0.57-0.99 | *0.02* | 0.76 | 0.58-1.02 | *0.04* |
|  | Best vs Bad | 0.68 | 0.51-0.90 |  | 0.68 | 0.51-0.92 |  |
| **WHO stage** | 2 vs 1 | 1.09 | 0.87-1.36 | *0.69* | - | - |  |
|  | 3 & 4 vs 1 | 1.08 | 0.77-1.51 |  | - | - |  |
| **ART duration** | /12 Months | 1.00 | 0.91-1.10 | *0.91* | - | - |  |
| **CD4 (cells/mm^3^)** | ≤500 vs >500 | 1.09 | 0.89-1.33 | *0.37* | - | - |  |
| **Viral load (copies/ml)** | ≤5 vs >5 Log**_10_** | 0.93 | 0.76-1.14 | *0.50* | - | - |  |

**OR:** odds ratio; **aOR:** adjusted odds ratio; **CI:** confidence interval; **WHO:** World Health Organization; **ART:** antiretroviral therapy; **P:** p-value of the Logistic Model

*see Methods section for definition of living conditions
