# Supplementary material for: High correlation between Framingham equations with BMI and with lipids to estimate cardiovascular risks score at baseline in HIV-infected adults in the Temprano trial, ANRS 12136 in Côte d’Ivoire
Source: PLoS One. 2017 Jun 5;12(6):e0177440. doi: 10.1371/journal.pone.0177440 (PMC5459337; doi:10.1371/journal.pone.0177440)
Supplement: S4 Table — (DOCX) [file pone.0177440.s004.docx]

|  | | **Univariable analysis** | | | **Multivariable analysis** | | |
| --- | --- | --- | --- | --- | --- | --- | --- |
| **Variable** | **Unit** | **OR** | **CI_95%_** | *P* | **aOR** | **CI_95%_** | *P* |
| **Education level** | Primary vs Never | 0.95 | 0.59-1.51 | *0.59* | - | - |  |
|  | Secondary vs Never | 1.00 | 0.64-1.55 |  | - | - |  |
|  | Superior vs Never | 0.66 | 0.35-1.25 |  | - | - |  |
| **Employment** | Public/Private vs No activity | 2.02 | 1.29-3.17 | *0.0005* | 1.99 | 1.25-3.16 | *0.0005* |
|  | Informal vs No activity | 1.00 | 0.64-1.56 |  | 0.93 | 0.59-1.46 |  |
| **Matrimonial status** | Married vs Single | 3.42 | 2.20-5.32 | *0.0001* | 3.26 | 2.09-5.09 | *0.0001* |
|  | Divorced vs Single | 3.93 | 2.22-6.96 |  | 4.33 | 2.42-7.73 |  |
| **Living conditions*** | Moderate vs Bad | 0.98 | 0.60-1.59 | *0.96* | - | - | *-* |
|  | Best vs Bad | 0.94 | 0.57-1.54 |  | - | - |  |
| **WHO stage** | 2 vs 1 | 1.67 | 1.16-2.42 | *0.01* | 1.69 | 1.16-2.46 | *0.01* |
|  | 3 & 4 vs 1 | 0.81 | 0.41-1.61 |  | 0.90 | 0.45-1.80 |  |
| **ART duration** | /12 Months | 0.99 | 0.98-1.03 | *0.91* | - | - |  |
| **CD4 (cells/mm3)** | ≤500 vs >500 | 1.061 | 0.71-1.43 | *0.93* | - | - |  |
| **Viral load (copies/ml)** | ≤5 vs >5 Log_10_ | 0.90 | 0.63-1.29 | *0.57* | - | - |  |

**S4 Table:** Association between patient baseline and therapeutic characteristics and moderate/high CV risk score at M30 in Temprano trial, Framingham with BMI, Abidjan (N=1700).

**OR:** odds ratio; **aOR:** adjusted odds ratio; **CI:** confidence interval; **WHO:** World Health Organization; **ART:** antiretroviral therapy; **P:** p-value of the Logistic Model

*see Methods section for definition of living conditions
